# A first-in-human, open-label multicentre Phase 1 study of the orally administered E7386 in patients with selected advanced neoplasms

**DOI:** 10.1038/s41416-026-03488-w

**Published:** 2026-06-04

**Authors:** T. R. Jeffry Evans, Natalie Cook, Anthony El-Khoueiry, David J. Pinato, Nguyen H. Tran, David Hsiehchen, Edward Mena, Tim Meyer, Jincao Wu, Shriram M. Pathak, Costanza Paoletti, Lea Dutta, Chinyere E. Okpara, Juanita S. Lopez

**Affiliations:** 1 University of Glasgow, Beatson West of Scotland Cancer Centre, Glasgow, UK; 2 Department of Medical Oncology, The Christie NHS Foundation Trust, Manchester, UK; 3 Division of Cancer Sciences, University of Manchester, Manchester, UK; 4 Division of Medical Oncology, Keck School of Medicine, University of Southern California, Norris Comprehensive Cancer Center, Los Angeles, CA USA; 5 Department of Surgery & Cancer, Imperial College London, London, UK; 6 Department of Translational Medicine, Division of Oncology, University of Piemonte Orientale, Novara, Italy; 7 Department of Oncology, Mayo Clinic Comprehensive Cancer Center, Rochester, MN USA; 8 Division of Hematology and Oncology, UT Southwestern Medical Center, Dallas, TX USA; 9 Pasadena Liver Center, California Liver Research Institute, Pasadena, CA USA; 10 Research Department of Oncology, UCL Cancer Institute, University College London, London, UK; 11 Department of Oncology, Royal Free Hospital, London, UK; 12 Biostatics, Eisai Inc., Nutley, NJ USA; 13 Clinical Pharmacology Science, Eisai Ltd, Hatfield, UK; 14 Clinical Research, Eisai Inc., Nutley, NJ USA; 15 Clinical Development, Eisai Ltd, Hatfield, UK; 16 Drug Development Unit, The Institute of Cancer Research and The Royal Marsden Hospital NHS Foundation Trust, London, UK

**Keywords:** Oncology, Cancer

## Abstract

**Background:**

We present data from the Phase 1 open-label Study 101 of E7386, an oral protein-protein interaction inhibitor reported to block the CBP/β-catenin interaction, in patients with solid tumours.

**Methods:**

Eligible patients (across the UK and US) aged ≥18 years had advanced/recurrent solid tumours (dose-escalation) or *CTNNB1*-mutated hepatocellular carcinoma (dose-expansion). Primary objectives were to assess safety/tolerability and determine the recommended Phase 2 dose (RP2D) of E7386.

**Results:**

Thirty-eight patients received study drug (dose-escalation: *n* = 32; expansion: *n* = 6; dose-range: 5–120 mg twice daily [BID]); 60.5% received ≥3 prior anticancer medications. Dose-limiting toxicities (grade-2 lethargy and grade-2 decreased appetite) occurred in 1 patient (20 mg BID cohort). The RP2D was determined as 120 mg BID. Most (94.7%) patients experienced treatment-related adverse events (TRAEs), most frequently nausea (65.8%) and vomiting (60.5%), which were primarily grade 1/2 and well-managed with antiemetics. No grade 4/5 TRAEs were noted. Pharmacokinetic exposure increased with increasing dose, although large intersubject variability was observed. No objective responses were noted; stable disease (SD) was observed in 36.8% of patients, including SD ≥ 23 weeks in 18.8% of patients (dose-escalation).

**Conclusions:**

E7386 demonstrated a manageable safety profile, a dose-dependent pharmacokinetic profile, and some disease stabilisation in heavily pretreated patients with advanced solid tumours.

**Trial registration:**

NCT03264664 https://clinicaltrials.gov/study/NCT03264664.

## Introduction

The Wnt/β-catenin signalling pathway plays a pivotal role in governing the cell cycle, stem cell renewal, proliferation, and differentiation. In the nucleus, β-catenin forms a transcriptional complex with TCF/LEF transcription factors and the co-activator CBP (cAMP response element binding protein [CREB]-binding protein) [1, 2]. This β-catenin-TCF/LEF-CBP complex drives the transcription of downstream target genes of the Wnt/β-catenin pathway. Aberrant activation of the Wnt/β-catenin pathway results in inhibition of the degradation of β-catenin (encoded by *CTNNB1*), which can lead to transcription dysregulation of several genes, including those associated with the development of cancers, thereby contributing to carcinogenesis [3–5].

Genetic alterations in the Wnt/β-catenin pathway play a notable role in several neoplasms, including hepatocellular carcinoma (HCC), colorectal cancer, desmoid tumours, and endometrial cancer. In HCC, the frequency of *CTNNB1* mutations is high [6], and other oncogenic driver gene mutations rarely co-occur, making targeting the Wnt/β-catenin pathway an attractive strategy [7]. Currently, treatment options targeting the Wnt/β-catenin pathway are limited and not well established for cancer [5].

E7386 is an orally administered protein-protein interaction inhibitor reported to block the interaction between CBP and β-catenin, resulting in modulation of Wnt/β-catenin pathway-dependent gene expression. In preclinical studies, E7386 suppressed polyp formation in the intestines of *Apc*^*Min/+*^ mice and significantly inhibited in vitro and in vivo tumour growth in an ECC10, *APC*-mutated human gastric cancer model, as well as in an MMTV-Wnt1 mouse breast cancer model with activation of the Wnt/β-catenin pathway by overexpression of *Wnt1* [8]. Furthermore, E7386’s mechanism of action appears to extend beyond the Wnt/β-catenin pathway [9, 10]; E7386 has also been reported to upregulate the activating transcription factor 4 (ATF4) pathway and activate a cellular integrated stress response in preclinical models of HCC [9].

In the Dose Escalation Part and Expansion Part 1 of the Phase 1 Study 103 (conducted in 55 patients in Japan), E7386 monotherapy demonstrated a manageable safety profile and a dose-dependent pharmacokinetic (PK) profile in heavily pretreated patients with advanced solid tumours. Two patients had partial responses (1 had small bowel carcinoma, and 1 had a desmoid tumour) [11].

Here, we present the results of a first-in-human, open-label, multicentre, Phase 1 Study 101, conducted in a Western population (sites located in the United States and the United Kingdom) to complement the data obtained from Asian patients in Study 103. The safety, tolerability, PK and antitumour activity of ascending doses of E7386 were investigated in patients with selected advanced or recurrent neoplasms.

## Methods

### Study design

This multicentre, open-label, Phase 1 study (clinicaltrials.gov identifier: NCT03264664) was conducted to assess the safety and tolerability (including determination of recommended Phase 2 dose [RP2D]) of E7386 monotherapy in patients with solid tumours (Supplementary Fig. 1). The study included an initial Dose Escalation Part (referred to as the Escalation Part), conducted at sites across the United Kingdom, in patients with selected tumour types. The Dose Escalation Part was followed by a Dose Expansion Part (referred to as the Expansion Part), conducted at sites across the United Kingdom and the United States, in patients with HCC whose tumour was known to harbour *CTNNB1* gain-of-function mutations as detected either in tumour tissue or circulating tumour DNA (ctDNA) using a Sponsor-approved assay. In the Escalation Part, dose levels were assigned using a modified toxicity probability interval dose escalation methodology, which allows for efficient escalation to higher dose levels, much faster with as few as 2 patients per dose level in the case of a favourable toxicity profile [12]. The Expansion Part was to be initiated after completion of the Escalation Part, as a result of achieving a maximum tolerated dose (MTD), selection of the RP2D, or after a decision was made to evaluate more than 1 potential RP2D level in the Expansion Part. The objective was to further evaluate the safety and preliminary efficacy of E7386 at the RP2D and to further characterise PK and pharmacodynamics.

The initial dose was 5 mg twice daily (BID) as a capsule formulation up to 45 mg BID (5 mg, 10 mg, 20 mg, 30 mg, and 45 mg BID) after which the formulation was updated from capsule to tablet (immediate release) from 45 mg BID (45 mg, 80 mg, 100 mg, and 120 mg BID); study drug was administered continuously in 28-day cycles. The dose was escalated in the next cohort if no dose-limiting toxicities (DLTs) were observed in the previous cohort.

### Patients

Eligible patients were at least 18 years old with advanced or recurrent tumour types (confirmed by available histology/cytology records) who required systemic therapy, and for whom no alternative standard therapy was available. In the Escalation Part, tumour types included those reported to have higher incidence of Wnt pathway mutations (such as *APC* or *CTNNB1*) expected to be sensitising for E7386 as a monotherapy treatment: desmoid tumours, anaplastic thyroid cancer, endometrial cancer, melanoma, colorectal cancer, HCC, pancreatic cancer, prostate cancer, ovarian cancer, head and neck cancer, or additional tumour types (such as adrenal cortical carcinoma, on discussion and agreement with the sponsor). In the Expansion Part, patients were required to have HCC with confirmed *CTNNB1* gain-of-function mutations, per tumour tissue biopsy or ctDNA analysis using a Sponsor-approved assay, which may have included locally or centrally approved assays. In the Expansion Part, patients must have also had: (1) a diagnosis of HCC that was histologically confirmed or clinically confirmed (excluding fibrolamellar, sarcomatoid, or mixed cholangio-HCC tumours) according to the American Association for the Study of Liver Disease criteria, including cirrhosis of any aetiology and/or chronic hepatitis B or C infection; and (2) Barcelona Clinic Liver Cancer Stage C disease, or Barcelona Clinic Liver Cancer Stage B disease not suitable for locoregional therapy or refractory to locoregional therapy and not suitable for a potentially curative treatment approach.

Additional inclusion criteria for both the Escalation and Expansion Parts included at least 1 measurable lesion based on RECIST v1.1, using computed tomography (CT)/magnetic resonance imaging (MRI); Eastern Cooperative Oncology Group (ECOG) performance status (PS) of 0–1 and life expectancy of at least 12 weeks; and adequate organ function (liver, kidney, bone marrow) and serum mineral levels. Patients with bone disease/conditions (eg, diagnosed osteoporosis with a T-score of <−2.5 by dual energy X-ray absorptiometry [DXA] scan or bone metastasis not treated with a bisphosphonate or denosumab, unless such treatment was started at least 14 days before cycle 1 day 1 or previous solitary bone lesions controlled with radiotherapy) were not eligible (per reasons detailed below). Significant cardiovascular impairment, including prolonged QT/QTc interval (QTc > 450 ms), was not permitted. In the Expansion Part, patients with HCC were excluded if they had: a Child Pugh status of B/C or clear invasion of HCC into the bile duct or portal vein invasion of Vp4 or symptomatic gastric or oesophageal varices per investigator’s clinical judgement or had a history of hepatic encephalopathy within 6 months before starting study drug.

### Endpoints

Primary endpoints were to determine the safety/tolerability profile of E7386, including MTD and/or RP2D of E7386. Secondary endpoints were to evaluate objective response rate and progression-free survival (PFS) in patients who received E7386, and to evaluate the PK profile of E7386.

### Assessments

Tumour response was assessed using CT/MRI scans, per RECIST v1.1, at screening and then every 8 weeks. Bone scans (^99^m-technetium polyphosphate scintigraphy, whole-body bone MRI, or ^18^F-NaF) were performed at screening, every 24 weeks (±2 weeks) in conjunction with a scheduled tumour assessment visit, and as clinically indicated, and within 1 week after confirmation of complete response. Bone scans were not required for patients with desmoid tumour (Escalation Part) and HCC (Expansion Part). A brain scan (CT or MRI) was required to be performed at screening to assess potential central nervous system disease and/or metastases. For patients with previously treated eligible brain metastases, a brain scan was required to be performed at all tumour assessment time points. For HCC (Expansion Part only), triphasic imaging of the liver (either by CT or MRI) was required to be performed in addition to contrast-enhanced CT of the chest and contrast-enhanced CT or MRI of the abdomen, pelvis, and other areas of known disease at screening.

Safety assessments consisted of monitoring and recording adverse events (AEs) of all severity grades, including DLTs; AEs were graded according to the Common Terminology Criteria for Adverse Events of the National Cancer Institute version 4.03 from the time of informed consent until 30 days after the last dose or until the patient initiated a new anticancer therapy, whichever was sooner. DLTs were defined as AEs related to E7386 occurring during cycle 1 (28 days) and are listed in the Supplementary Information.

Treatment-related vomiting was to be treated with a serotonin (5-HT_3_) receptor antagonist such as ondansetron. Prophylactic administration of antiemetics was recommended after the completion of the Escalation Part of the study and was not recommended during the DLT assessment period to fully capture the true incidence of these AEs. Administration of antiemetics was, however, allowed partway through treatment.

Because bone fragility fractures have been observed in studies with other agents targeting the Wnt signalling pathway [13, 14], and bone toxicity has been noted with E7386 in preclinical studies, serial bone densitometry assessments were conducted in this trial to monitor and mitigate the risk of bone toxicity. Bone densitometry (DXA) of the hip and spine (and, where possible, the radius) was performed at 12 (±2) week intervals, and T-scores were assessed by the investigator for decreases in bone density. Decreases of T-scores to −2.5 or more necessitated patients taking antiresorptive agents.

Given that the Wnt signalling pathway plays a pivotal role in ocular vascular development [15], modulation of this pathway poses a theoretical risk for ocular toxicity. Thus, ophthalmological assessments, including visual acuity and direct/indirect ophthalmoscopy, were performed at screening/baseline and at the first follow-up at 3 months (±7 days) and then every 6 months (±7 days), thereafter, if no toxicity was identified. If patients reported symptoms of visual disturbances, these were to be managed according to local or institutional guidelines in conjunction with specialist consultation.

Blood samples for PK analyses were collected during cycle 1 on days 1 and 8 predose (0 h), and at 0.5, 1, 2, 4, 6, 8, 10, and 12 h postdose in the Escalation Part. Additionally, on days 15 and 22, a predose blood sample was collected for PK analysis. Urine samples for PK analyses were collected during the Dose Escalation Part of the study in cycle 1 only. For each patient, all urine excreted was collected at the following time points: cycle 1 day 1 pre‑dose (0 h), and post‑dose intervals of 0–6 h, 6–12 h, and 12–24 h on cycle 1 day 1 and cycle 1 day 8. In the Expansion Part, blood samples for PK analyses were collected predose and 1 h postdose on day 1 of cycles 1, 2, 3, and 4. PK parameters were derived by noncompartmental analysis using actual time points.

### Statistical methods

Statistical analyses were performed using SAS version 9.4 (SAS Institute, Cary, NC, USA) or higher. The modified toxicity probability interval dose escalation methodology was used to determine the MTD and/or RP2D of E7386.

The DLT analysis set for evaluating tolerability included all patients in the Escalation Part who had completed cycle 1, without major protocol deviation (that affected the DLT assessment), had at least 75% of study drug compliance, and were evaluable for DLT, or patients who had experienced a DLT during cycle 1. The full analysis set (baseline characteristics and efficacy) or safety analysis set (all safety except DLTs) included all patients who received at least 1 dose of the study drug.

The PK analysis set included all patients who received at least 1 dose of the study drug and had at least 1 evaluable plasma concentration. PK parameters were derived using noncompartmental analysis based on actual time points collected during the Escalation Part. Summary statistics were tabulated for the PK parameters of E7386 by part, dose level, and day.

The objective response rate was calculated with an exact 95% CI using the Clopper-Pearson method. PFS and overall survival were summarised using Kaplan-Meier estimates.

## Results

### Patients

Between 27 July 2017 and the data cutoff date for primary analysis (20 May 2024), 58 patients signed the informed consent form, and 20 patients failed screening. After screening, a total of 38 patients received study treatment.

In the Escalation Part, 32 patients received BID E7386 with E7386 5 mg capsule (*n* = 2), 10 mg capsule (*n* = 2), 20 mg capsule (*n* = 10), 30 mg capsule (*n* = 2), 45 mg capsule (*n* = 2), 45 mg tablet (*n* = 2), 80 mg tablet (*n* = 2), 100 mg tablet (*n* = 6), and 120 mg tablet (*n* = 4). In the Expansion Part, 6 patients with HCC received E7386 120 mg tablet BID (RP2D determined in the Escalation Part).

The DLT analysis set in the Escalation Part included 28 patients (4 patients did not complete treatment cycle 1). The full analysis, safety analysis, and PK analysis sets each included 38 patients: 32 patients from the Escalation Part and 6 patients from the Expansion Part. By the data cutoff date, in the Escalation Part, 31 (96.9%) patients discontinued treatment. One (3.1%) patient with a desmoid tumour in the 30 mg capsule treatment group was still receiving treatment (ongoing after 63.2 months). The primary reasons for discontinuation were radiological or clinical disease progression (23 [71.9%] and 3 [9.4%] patients, respectively) (Supplementary Fig. 2). Additional reasons for treatment discontinuation were AEs (3 [9.4%] patients), patient choice, and withdrawal of consent (1 patient each [3.1%]). In the Expansion Part, all 6 patients discontinued treatment due to radiological or clinical disease progression (4 [66.7%] and 2 [33.3%] patients, respectively).

### Baseline characteristics

Baseline disease history and characteristics are presented in Table 1. Overall, the median age of patients was 59.0 (range: 30–79) years; most patients were <65 years old (*n* = 23, 60.5%), male (*n* = 24, 63.2%), White (*n* = 34, 89.5%), non-Hispanic or Latino (*n* = 36, 94.7%), and had an ECOG PS of 1 (*n* = 30, 78.9%). All 38 patients had received a prior anticancer regimen (either adjuvant/neoadjuvant, metastatic/locally advanced, unresectable, or unknown type; range: 1–7), and 23 patients (60.5%) were heavily pretreated with ≥3 anticancer regimens; most patients (76.3%) had prior anticancer treatment for metastatic/locally advanced, unresectable cancer. The overall median duration of the last anticancer regimen was 3.60 months (range: 0.72 to 23.72 months).

**Table 1:** Patient and disease baseline characteristics.

Category	Dose Escalation^a^										Dose Expansion^a^	Total (*n* = 38)
	5 mg capsule (*n* = 2)	10 mg capsule (*n* = 2)	20 mg capsule (*n* = 10)	30 mg capsule (*n* = 2)	45 mg capsule (*n* = 2)	45 mg tablet (*n* = 2)	80 mg tablet (*n* = 2)	100 mg tablet (*n* = 6)	120 mg tablet (*n* = 4)	Total (*n* = 32)	120 mg tablet (*n* = 6)	
**Median age, years (min-max)**^**b**^	66.0 **(**66.0–66.0)	61.0 (51.0–71.0)	56.0 (32.0–75.0)	49.5 (48.0–51.0)	38.5 (30.0–47.0)	50.5 (43.0–58.0)	71.0 (69.0–73.0)	59.5 (53.0–70.0)	59.5 (46.0–68.0)	58.0 (30–75)	69.5 (58.0–79.0)	59.0 (30.0–79.0)
**Age group,*n* (%)**												
<65 Years	0	1 (50.0)	7 (70.0)	2 (100)	2 (100)	2 (100)	0	4 (66.7)	3 (75.0)	21 (65.6)	2 (33.3)	23 (60.5)
≥65 years	2 (100)	1 (50.0)	3 (30.0)	0	0	0	2 (100)	2 (33.3)	1 (25.0)	11 (34.4)	4 (66.7)	15 (39.5)
**Sex,*n* (%)**												
Male	2 (100)	1 (50.0)	4 (40.0)	1 (50.0)	1 (50.0)	1 (50.0)	2 (100)	4 (66.7)	3 (75.0)	19 (59.4)	5 (83.3)	24 (63.2)
Female	0	1 (50.0)	6 (60.0)	1 (50.0)	1 (50.0)	1 (50.0)	0	2 (33.3)	1 (25.0)	13 (40.6)	1 (16.7)	14 (36.8)
**Race,*n* (%)**												
White	2 (100)	2 (100)	9 (90.0)	2 (100)	2 (100)	2 (100)	2 (100)	6 (100)	4 (100)	31 (96.9)	3 (50.0)	34 (89.5)
Black or African American	0	0	1 (10.0)	0	0	0	0	0	0	1 (3.1)	0	1 (2.6)
Asian	0	0	0	0	0	0	0	0	0	0 (0.0)	3 (50.0)	3 (7.9)
**Ethnicity,*n* (%)**												
Hispanic or Latino	0	0	0	1 (50.0)	0	0	0	0	0	1 (3.1)	1 (16.7)	2 (5.3)
Not Hispanic or Latino	2 (100)	2 (100)	10 (100)	1 (50.0)	2 (100)	2 (100)	2 (100)	6 (100)	4 (100)	31 (96.9)	5 (83.3)	36 (94.7)
**ECOG PS,*n* (%)**												
0	0	0	2 (20.0)	0	1 (50.0)	0	1 (50.0)	3 (50.0)	0	7 (21.9)	1 (16.7)	8 (21.1)
1	2 (100)	2 (100)	8 (80.0)	2 (100)	1 (50.0)	2 (100)	1 (50.0)	3 (50.0)	4 (100)	25 (78.1)	5 (83.3)	30 (78.9)
**Child-Pugh score,*n* (%)**												
5	–	–	–	–	–	–	–	–	–	–	4 (66.7)	–
6	–	–	–	–	–	–	–	–	–	–	2 (33.3)	–
**Alpha fetoprotein group,*n* (%)**												
<400 ng/mL	–	–	–	–	–	–	–	–	–	–	5 (83.3)	–
≥400 ng/mL	–	–	–	–	–	–	–	–	–	–	1 (16.7)	–
**Primary tumour histology/cytology,*n* (%)**												
Desmoid tumour	0 (0.0)	0 (0.0)	1 (10.0)	1 (50.0)	1 (50.0)	0 (0.0)	0 (0.0)	0 (0.0)	0 (0.0)	3 (9.4)	0 (0.0)	3 (7.9)
Colorectal cancer	2 (100)	1 (50.0)	4 (40.0)	1 (50.0)	0 (0.0)	2 (100)	1 (50.0)	5 (83.3)	3 (75.0)	19 (59.4)	0 (0.0)	19 (50.0)
Hepatocellular carcinoma	0 (0.0)	0 (0.0)	0 (0.0)	0 (0.0)	0 (0.0)	0 (0.0)	0 (0.0)	1 (16.7)	0 (0.0)	1 (3.1)	6 (100)	7 (18.4)
Pancreatic cancer	0 (0.0)	0 (0.0)	2 (20.0)	0 (0.0)	0 (0.0)	0 (0.0)	0 (0.0)	0 (0.0)	1 (25.0)	3 (9.4)	0 (0.0)	3 (7.9)
Melanoma	0 (0.0)	1 (50.0)	2 (20.0)	0 (0.0)	1 (50.0)	0 (0.0)	1 (50.0)	0 (0.0)	0 (0.0)	5 (15.6)	0 (0.0)	5 (13.2)
Adrenal cortical carcinoma	0 (0.0)	0 (0.0)	1 (10.0)	0 (0.0)	0 (0.0)	0 (0.0)	0 (0.0)	0 (0.0)	0 (0.0)	1 (3.1)	0 (0.0)	1 (2.6)
**Previous systemic anticancer therapy,*n* (%)**	2 (100)	2 (100)	10 (100)	2 (100)	2 (100)	2 (100)	2 (100)	6 (100)	4 (100)	32 (100)	6 (100)	38 (100)
**Number of previous anticancer medications,*n* (%)**												
0	0 (0.0)	0 (0.0)	0 (0.0)	0 (0.0)	0 (0.0)	0 (0.0)	0 (0.0)	0 (0.0)	0 (0.0)	0 (0.0)	0 (0.0)	0 (0.0)
1	0 (0.0)	0 (0.0)	3 (30.0)	1 (50.0)	0 (0.0)	0 (0.0)	0 (0.0)	0 (0.0)	0 (0.0)	4 (12.5)	2 (33.3)	6 (15.8)
2	0 (0.0)	0 (0.0)	3 (30.0)	0 (0.0)	0 (0.0)	0 (0.0)	2 (100)	1 (16.7)	1 (25.0)	7 (21.9)	2 (33.3)	9 (23.7)
3	0 (0.0)	0 (0.0)	1 (10.0)	0 (0.0)	2 (100)	1 (50.0)	0 (0.0)	3 (50.0)	0 (0.0)	7 (21.9)	1 (16.7)	8 (21.1)
≥4	2 (100)	2 (100)	3 (30.0)	1 (50.0)	0 (0.0)	1 (50.0)	0 (0.0)	2 (33.3)	3 (75.0)	14 (43.7)	1 (16.7)	15 (39.5)
**Baseline mutation status,*n* (%)**^**c**^												
*APC*	1 (50.0)	0 (0.0)	0 (0.0)	0 (0.0)	0 (0.0)	0 (0.0)	0 (0.0)	1 (16.7)	1 (25.0)	3 (9.4)	0 (0.0)	3 (7.9)
*CTNNB1*	1 (50.0)	0 (0.0)	1 (10.0)	1 (50.0)	0 (0.0)	1 (50.0)	0 (0.0)	0 (0.0)	0 (0.0)	4 (12.5)	6 (100)	10 (26.3)
*AXIN1*	0 (0.0)	0 (0.0)	0 (0.0)	0 (0.0)	0 (0.0)	0 (0.0)	0 (0.0)	0 (0.0)	0 (0.0)	0 (0.0)	0 (0.0)	0 (0.0)
*AXIN2*	0 (0.0)	0 (0.0)	0 (0.0)	0 (0.0)	0 (0.0)	0 (0.0)	0 (0.0)	0 (0.0)	0 (0.0)	0 (0.0)	0 (0.0)	0 (0.0)
*PTEN*	0 (0.0)	0 (0.0)	0 (0.0)	0 (0.0)	1 (50.0)	0 (0.0)	0 (0.0)	0 (0.0)	0 (0.0)	1 (3.1)	0 (0.0)	1 (2.6)
*PI3K*	0 (0.0)	0 (0.0)	0 (0.0)	0 (0.0)	0 (0.0)	0 (0.0)	0 (0.0)	0 (0.0)	0 (0.0)	0 (0.0)	0 (0.0)	0 (0.0)
*TP53*	1 (50.0)	0 (0.0)	1 (10.0)	0 (0.0)	0 (0.0)	1 (50.0)	0 (0.0)	1 (16.7)	2 (50.0)	6 (18.8)	1 (16.7)	7 (18.4)
*FGFR2*	0 (0.0)	0 (0.0)	0 (0.0)	0 (0.0)	0 (0.0)	0 (0.0)	0 (0.0)	0 (0.0)	0 (0.0)	0 (0.0)	0 (0.0)	0 (0.0)
*BRAF*	0 (0.0)	1 (50.0)	0 (0.0)	0 (0.0)	1 (50.0)	0 (0.0)	0 (0.0)	0 (0.0)	0 (0.0)	2 (6.3)	0 (0.0)	2 (5.3)
*NRAS*	0 (0.0)	0 (0.0)	0 (0.0)	0 (0.0)	0 (0.0)	0 (0.0)	1 (50.0)	0 (0.0)	0 (0.0)	1 (3.1)	0 (0.0)	1 (2.6)
*KRAS*	1 (50.0)	0 (0.0)	3 (30.0)	0 (0.0)	0 (0.0)	0 (0.0)	0 (0.0)	3 (50.0)	2 (50.0)	10 (31.3)	0 (0.0)	10 (26.3)

*APC* adenomatous polyposis coli, *AXIN* axin, *BRAF* serine/threonine-protein kinase B-Raf, *ECOG* Eastern Cooperative Oncology Group, *PS* performance status, *FGFR2* fibroblast growth factor receptor 2, *max* maximum, *min*, minimum, *PI3K* phosphoinositide 3-kinase, *PTEN* phosphatase and tensin homologue, *TP53* tumour protein p53.

^a^E7386 was administered twice daily in all dose levels/formulations.

^b^Age is calculated at the date of informed consent.

^c^As reported by the study site (per circulating tumour DNA or tumour tissue); in the Dose Expansion Part only, *CTNNB1* mutations were detected either in tumour tissue or circulating tumour DNA by a Sponsor-approved assay. Patients could have had more than one mutation at baseline.

In the Escalation Part, the median age of patients was 58.0 (range: 30–75) years; most patients (*n* = 21, 65.6%) were <65 years old, male (*n* = 19, 59.4%), White (*n* = 31, 96.9%), and had an ECOG PS of 1 (*n* = 25, 78.1%). Nineteen (59.4%) patients had colorectal cancer, 5 (15.6%) patients had melanoma, 3 (9.4%) patients each had desmoid tumours or pancreatic cancer, and 1 (3.1%) patient each had HCC or adrenal cortical carcinoma. Patients were heavily pretreated (21 [65.6%] received ≥3 lines of treatment); most had received prior treatment with oxaliplatin (68.8%), irinotecan (65.6%), fluorouracil (62.5%), capecitabine (43.8%), and tipiracil;trifluridine (31.3%).

In the Expansion Part, the median age of patients was 69.5 (range: 58–79) years; most patients (*n* = 4, 66.7%) were ≥65 years old, male (*n* = 5, 83.3%), White (*n* = 3, 50.0%) or Asian (*n* = 3, 50.0%), and had an ECOG PS of 1 (*n* = 5, 83.3%; Table 1). Alpha fetoprotein at study entry was <400 ng/mL in 5 (83.3%) patients. Four patients (66.7%) were previously treated with ≥2 lines of therapy, including one with 3 and another with 4 prior lines of therapy. Five patients (83.3%) had received prior anti-PD-L1 treatment (most frequently atezolizumab [83.3%]), the remaining patient had contraindications. All patients had received anti-angiogenesis treatment: 83.3% had received prior bevacizumab, 66.7% had received lenvatinib, and 33.3% had received cabozantinib.

If available, historical data of tumour mutation status were recorded at study entry. Ten patients (26.3%; 4 in the Escalation Part and all 6 in the Expansion Part) had tumours harbouring a *CTNNB1* mutation; of those patients, 8 had an oncogenic alteration, 1 had a variant of unknown significance, and 1 had no details available regarding their mutation. Ten patients (26.3%; all in the Escalation Part) had tumours harbouring a known *KRAS* mutation, and 7 patients (18.4%; 6 in the escalation and 1 in the Expansion Part) had a known *TP53* mutation. Three patients (7.9%; all in the Escalation Part) had a known *APC* mutation, 2 patients (5.3%; both in the Escalation Part) had a *BRAF* mutation, 1 patient (2.6%; in the Escalation Part) had a *PTEN* mutation, and 1 patient (2.6%; in the Escalation Part) had a known *NRAS* mutation (Table 1).

### Dose-limiting toxicities

Of the 28 patients included in the DLT analysis set, 1 patient in the E7386 20 mg BID capsule group experienced grade 2 lethargy and grade 2 decreased appetite, which met DLT criteria (ie, grade 2 non-haematologic treatment-related events). The study drug was initially interrupted and later reduced due to these events, as well as due to a treatment-emergent adverse event of nausea; the patient discontinued the study due to radiological disease progression. At the time of discontinuation, the event of lethargy was reported as resolving, and the event of decreased appetite had resolved. No patients in other dose groups experienced DLTs. The E7386 120 mg BID dose level was deemed tolerable and was determined to be the RP2D of E7386 as monotherapy.

### Safety

#### Treatment-emergent and treatment-related adverse events

Thirty-eight (100%) patients experienced at least 1 treatment-emergent adverse event, and a summary of treatment-emergent adverse events is reported in Supplementary Table 1. Thirty-six (94.7%) patients experienced at least 1 treatment-related adverse event (TRAE) as determined by the investigator. The most frequent (>15% of overall; Table 2) TRAEs assessed as related to treatment by the investigator were: nausea (65.8%), vomiting (60.5%), fatigue (28.9%), and decreased appetite and diarrhoea (15.8% each). Among the 36 patients with TRAEs, most (*n* = 29/36; 80.6%) were grade 1 and 2, primarily consisting of gastrointestinal TRAEs, including nausea (*n* = 24/36; 66.7%) and vomiting (*n* = 22/36; 61.1%). Among all patients (*n* = 38), grade ≥3 TRAEs occurred in 7 (18.4%) patients (worst grade per patient, all cycles). Two patients had grade 3 treatment-related hypertension; and 1 patient each had grade 3 treatment-related abdominal infection, anaemia, cholelithiasis, dyspnoea, nausea, vomiting, white blood cell count decreased, and electrocardiogram QT prolonged. Of note, grade 3 QT prolongation occurred in a patient dosed at 100 mg BID who recovered after 2 days, but discontinued treatment/study due to their choice. No grade 4 or 5 TRAEs were reported. Most patients received antiemetics and antinauseants (*n* = 29; 76.3%) as concomitant medications. The majority of patients (65.8%) received ondansetron (which belongs to 5-HT_3_ antagonist subclass), 23.7% received cyclizine, and 21.1% received metoclopramide.

**Table 2:** Treatment-related adverse events occurring in >15% of overall patients in the Dose Escalation Part and Dose Expansion Part.

	Dose Escalation										Dose Expansion	Total (*n* = 38)
Category	5 mg capsule (*n* = 2)	10 mg capsule (*n* = 2)	20 mg capsule (*n* = 10)	30 mg capsule (*n* = 2)	45 mg capsule (*n* = 2)	45 mg tablet (*n* = 2)	80 mg tablet (*n* = 2)	100 mg tablet (*n* = 6)	120 mg tablet (*n* = 4)	Total (*n* = 32)	120 mg tablet (*n* = 6)	
**TRAEs,*n* (%)**	2 (100)	2 (100)	10 (100)	2 (100)	2 (100)	2 (100)	2 (100)	5 (83.3)	3 (75.0)	30 (93.8)	6 (100)	36 (94.7)
Nausea	1 (50.0)	2 (100)	6 (60.0)	2 (100)	2 (100)	1 (50.0)	1 (50.0)	4 (66.7)	2 (50.0)	21 (65.6)	4 (66.7)	25 (65.8)
Vomiting	0 (0.0)	2 (100)	6 (60.0)	2 (100)	1 (50.0)	1 (50.0)	1 (50.0)	3 (50.0)	2 (50.0)	18 (56.3)	5 (83.3)	23 (60.5)
Fatigue	0 (0.0)	0 (0.0)	3 (30.0)	1 (50.0)	0 (0.0)	1 (50.0)	0 (0.0)	2 (33.3)	1 (25.0)	8 (25.0)	3 (50.0)	11 (28.9)
Decreased appetite	0 (0.0)	1 (50.0)	4 (40.0)	0 (0.0)	0 (0.0)	0 (0.0)	0 (0.0)	0 (0.0)	1 (25.0)	6 (18.8)	0 (0.0)	6 (15.8)
Diarrhoea	0 (0.0)	0 (0.0)	0 (0.0)	0 (0.0)	0 (0.0)	0 (0.0)	0 (0.0)	2 (33.3)	2 (50.0)	4 (12.5)	2 (33.3)	6 (15.8)
**TRAE: worst CTCAE grade of** **≥** **3,*n*** (**%)**	0 (0.0)	0 (0.0)	3 (30.0)	0 (0.0)	0 (0.0)	0 (0.0)	0 (0.0)	1 (16.7)	1 (25.0)	5 (15.6)	2 (33.3)	7 (18.4)
**Serious TRAEs**^**a**^,***n*** (**%)**	0 (0.0)	0 (0.0)	2 (20.0)	0 (0.0)	0 (0.0)	0 (0.0)	0 (0.0)	0 (0.0)	1 (25.0)	3 (9.4)	0 (0.0)	3 (7.9)
**TRAEs leading to study drug discontinuation,*n*** (**%)**	0 (0.0)	0 (0.0)	0 (0.0)	0 (0.0)	0 (0.0)	0 (0.0)	0 (0.0)	0 (0.0)	1 (25.0)	1 (3.1)	1 (16.7)	2 (5.3)
**TRAEs leading to study drug dose reduction,*n* (%)**	0 (0.0)	0 (0.0)	1 (10.0)	0 (0.0)	0 (0.0)	0 (0.0)	0 (0.0)	0 (0.0)	0 (0.0)	1 (3.1)	2 (33.3)	3 (7.9)
**TRAEs leading to study drug interruption,*n* (%)**	0 (0.0)	0 (0.0)	4 (40.0)	1 (50.0)	0 (0.0)	0 (0.0)	0 (0.0)	1 (16.7)	2 (50.0)	8 (25.0)	2 (33.3)	10 (26.3)

*CTCAE* Common Terminology Criteria for Adverse Events, *TRAE* treatment-related adverse event.

^a^Patients may be counted in multiple categories.

TRAEs led to dose interruption of E7386 (Table 2) in 10 (26.3%) patients overall, 8 (25.0%) in the Escalation Part, and 2 (33.3%) in the Expansion Part. TRAEs led to dose reduction in 3 (7.9%) patients overall: 1 (3.1%) patient in the Escalation Part (grade 2 lethargy, grade 2 nausea, and grade 2 decreased appetite) and 2 (33.3%) patients in the Expansion Part (1 patient experienced grade 1 fatigue, grade 3 nausea, and grade 2 vomiting; 1 patient experienced grade 2 neutrophil count decreased, and grade 3 white blood cell decreased). TRAEs led to treatment discontinuation in 2 (5.3%) patients overall, 1 (3.1%) patient in the Escalation Part (grade 3 abdominal infection), and 1 patient (16.7%) in the Expansion Part (grade 2 fatigue). A total of 30 (78.9%) deaths were reported overall; none of the fatal AEs were considered related to E7386.

#### Bone health disorders and ocular disorders

Overall, few bone health disorders were observed among the 38 patients; 1 (2.6%) patient receiving E7386 10 mg BID experienced a grade 2 pathological fracture, which was considered a serious AE; this was not deemed treatment-related by investigators, and no action was taken regarding the study drug.

Ocular disorder events occurred in 4 (10.5%) patients. One patient in the 10 mg BID dose group of the Escalation Part experienced a grade 1 eyelid rash, which was considered not serious but was treatment-related; no action was taken regarding E7386. One patient in the 20 mg BID dose group of the Escalation Part had grade 1 visual impairment (not serious AE, considered treatment-related), which was resolved on the same day without treatment; no action was taken regarding the study drug for visual impairment. Later, this patient also experienced grade 3 hemianopia (serious AE, considered not treatment-related), for which E7386 was interrupted. The patient then underwent tumour assessment on the same day, which showed radiologic disease progression, including brain metastases, after which study treatment was discontinued due to disease progression. One patient in the 20 mg BID dose group of the Escalation Part had grade 1 photophobia, which was not serious, and considered treatment-related; no action was taken regarding E7386 for photophobia. One patient in the 120 mg BID dose group of the Expansion Part had grade 1 diplopia, grade 1 vitreous detachment, and grade 2 cataract and blurred vision, which were not serious. All 4 AEs were considered treatment-related, leading to temporary interruption of E7386 treatment; no other supportive medical intervention was used. Subsequently, diplopia resolved, and direct ophthalmologic assessment showed no worsening from the previous evaluation. Indirect ophthalmoscopy examination of both eyes was normal, and both blurred vision and cataract improved to grade 1.

### PK analysis

Mean (+standard deviation) plasma concentration-time curves of E7386 after dosing on cycle 1 day 1 and cycle 1 day 8 are presented on linear scales in Supplementary Fig. 3. E7386 plasma concentrations generally increased with increasing doses of E7386 (5–120 mg) after both first (cycle 1 day 1) and repeated twice-daily administrations (cycle 1 day 8). However, large intersubject variability was observed (Table 3).

**Table 3:** Pharmacokinetic parameters of E7386 in the Dose Escalation Part: cycle 1—day 1 and day 8.

E7386 dose, mg twice daily	Cycle 1 day 1				Cycle 1 day 8					
	C_max_ (ng/mL)	t_max_ (h)	AUC_(0–12 h)_ (h*ng/mL)	Fe (%)	C_max,ss_ (ng/mL)	t_max,ss_ (h)	AUC_(0-τ)_ (h*ng/mL)	R_ac_ (AUC)	R_ac_ (C_max_)	Fe (%)
5 (n = 2) capsule	22.6 (107)	0.590 (0.500–0.680)	63.6 (107)	2.13 (43.9)	21.7 (95.2)	1.55 (1.07–2.03)	95.7 (51.7)	1.51 (39.5)	0.961 (7.07)	2.35 (13.0)
10 (n = 2) capsule	35.0 (93.6)	1.52 (1.03–2.00)	150 (183)	1.15 (27.0)	58.7 (107)	1.50 (1.00–2.00)	240 (282)	1.60 (27.4)	1.68 (8.44)	1.54 (56.4)
20 (n = 10) capsule	72.4 (578)	1.10 (0.600–4.25)	343 (174)^a^	1.40 (183)	126 (220)	1.08 (0.520–8.18)	403 (248)	1.73 (37.0)^b^	1.74 (102)	2.07 (130)^a^
30 (n = 2) capsule	35.9 (86.0)	2.14 (2.07–2.20)	136 (34.5)	1.35 (19.0)	12.7 (80.0)	0.565 (0.00–1.13)	64.6 (85.3)	0.477 (148)	0.354 (268)	0.921 (3.15)
45 (n = 2) capsule	78.7 (504)	1.29 (0.550–2.02)	231 (168)	0.825 (52.5)	115 (212)	1.19 (1.17–1.20)	278 (131)	1.20 (16.5)	1.46 (53.8)	1.06 (20.6)
45 (n = 2) tablet	148 (8.63)	2.01 (1.92–2.10)	744 (28.5)	1.32 (59.0)	159 (33.9)	3.14 (2.10–4.17)	1110 (23.4)	1.50 (4.72)	1.08 (24.8)	1.94 (4.73)
80 (n = 2) tablet	323 (30.2)	3.08 (2.00–4.15)	1820 (29.6)	1.29 (15.4)	947 (62.4)	0.740 (0.480–1.00)	3100 (73.5)	1.71 (37.7)	2.93 (28.3)	2.24 (41.5)
100 (n = 6) tablet	439 (78.0)	1.50 (0.580–2.12)	1230 (39.2)^c^	2.07 (50.0)	725 (168)	0.850 (0.570–4.12)	2370 (95.7)	1.22 (44.6)^c^	1.65 (89.2)	2.50 (54.1)
120 (n = 4) tablet	765 (132)	2.01 (1.08–4.10)	3080 (121)	0.550 (198)	1330 (175)^d^	2.00 (0.600–10.1)^d^	5170 (102)^d^	2.43 (65.7)^d^	2.43 (102)^d^	1.92 (3.72)^d^

t_max_ is reported as median (range); all other values are reported as geometric mean (%CV). Noncompartmental analysis was conducted exclusively for the Dose Escalation Part of the PK assessment.

*AUC*, area under the plasma concentration-time curve; AUC_(0–12 h)_, area under the plasma concentration-time curve from zero time to 12 h after first dose on day 1; AUC_(0–τ)_, area under the plasma concentration-time curve at steady state after the first dose on day 8 from time zero to τ, where τ is the length of time of the dosing interval (τ = 12 h); C_max_, maximum observed plasma concentration after the first dose on day 1; C_max,ss_, maximum observed plasma concentration at steady state after the first dose on day 8; Fe, fraction of dose excreted in urine; R_ac_ (AUC), accumulation ratio calculated from AUC_(0–τ)_ and AUC_(0–12 h)_ after the first dose on day 1; R_ac_ (C_max_), accumulation ratio calculated from C_max,ss_ and C_max_; ss, steady state; t_max_, time to reach maximum (peak) plasma concentration after first dose on day 1; t_max,ss_, time to reach maximum (peak) plasma concentration at steady state after the first dose on day 8.

^a^*n* = 9.

^b^*n* = 8.

^c^*n* = 4.

^d^*n* = 3.

E7386 was absorbed rapidly after the first dose administration (cycle 1 day 1) with a median time to reach peak plasma concentration (t_max_) of 0.590 to 3.08 h and 0.565 to 3.14 h after repeated twice-daily administration on cycle 1 day 8 (Table 3). Geometric means (% coefficient of variation) of accumulation ratios of E7386 based on area under the plasma concentration-time curve (AUC), and the maximum observed plasma concentration (C_max_) on day 8 ranged from 0.477 to 2.43 and 0.354 to 2.93, respectively. At cycle 1 day 8, the geometric mean fraction of E7386 excreted (%Fe) in urine as unchanged E7386 over 24 h ranged from 0.921% to 2.50% of the dose, indicating that excretion via urine was a minor pathway of E7386 elimination. Pharmacodynamic analyses will be presented separately and pooled with other studies of E7386.

### Antitumour activity

**Fig. 1:**
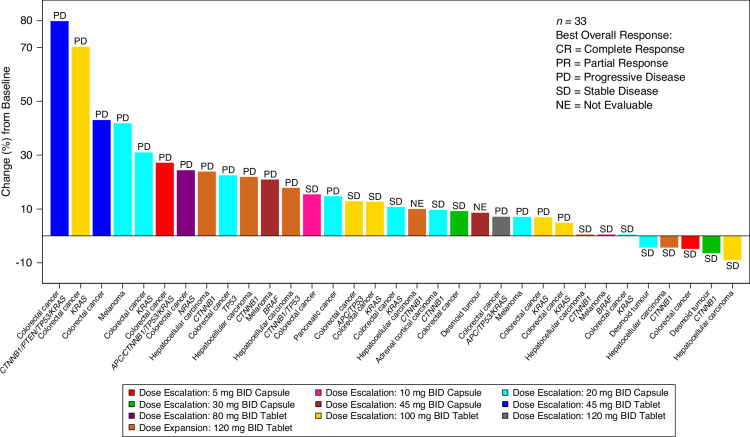
Percentage change in the sum of diameters of target lesions from baseline to post-baseline nadir. This figure includes patients (*n*) with both baseline and at least 1 post-baseline target lesion assessment. At the time of the data cutoff, 4 patients had a baseline target lesion assessment only, and 1 patient with PD did not have maximum tumour shrinkage and was excluded from the waterfall plot, as they had a new lesion. Patients with NE had post-baseline tumour assessment and target lesion diameter assessment. However, the tumour assessment was done <7 weeks, so they are denoted as NE given early SD (<7 weeks), based on RECIST v1.1. Gene names noted next to cancer type represent mutation(s) in *CTNNB1*, *PTEN*, *TP53*, *APC*, *KRAS*, *AXIN1*, *AXIN2*, *PI3K*, *FGFR2*, *BRAF*, or *NRAS-*based mutation status determined at baseline. BID twice daily, CR complete response, NE not evaluable, PD progressive disease, PR partial response, RECIST Response Evaluation Criteria in Solid Tumours, SD stable disease.

**Fig. 2:**
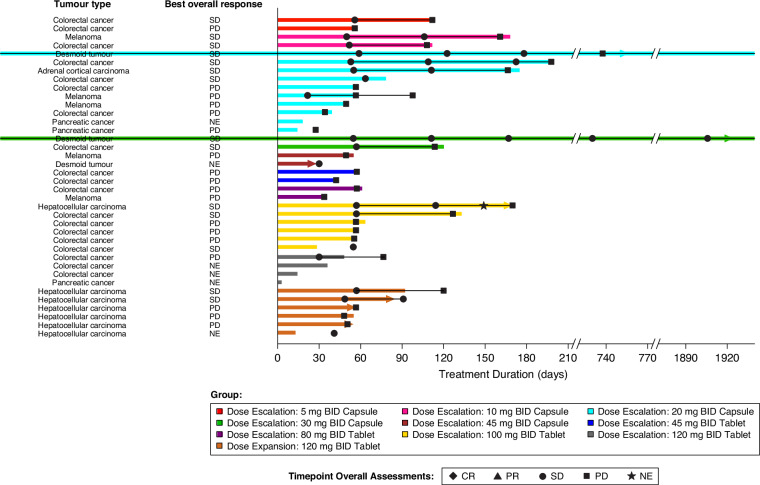
Duration of treatment and overall timepoint assessments in the Dose Escalation Part and the Dose Expansion Part. An arrow at the right edge of the bars indicates ongoing study at cutoff. Patients with NE, as best overall response, had early stable disease (<7 weeks). BID twice daily, CR complete response, NE not evaluable, PD progressive disease, PR partial response, SD stable disease.

A best overall response (BOR) of complete response or partial response was not observed in any patients in this study (Table 4). Percentage changes in the sums of diameters of target lesions from baseline to post-baseline nadir are shown in Fig. 1. Overall, stable disease (SD) was observed in 14 (36.8%) patients, 12 (37.5%) in the Escalation Part, and 2 (33.3%) in the Expansion Part. For these patients with a BOR of SD, the median duration of SD was 5.4 months (95% CI: 3.5–6.5) in the Escalation Part and 3.9 months (95% CI not evaluable–not evaluable) in the Expansion Part. SD lasted ≥23 weeks in 6 (18.8%) patients in the Escalation Part (1 patient each with melanoma, colorectal cancer, adrenal cortical carcinoma, and HCC; 2 patients with a desmoid tumour), and in no patient in the Expansion Part (Fig. 2).

**Table 4:** Tumour response and survival in the Dose Escalation Part and the Dose Expansion Part.

	Dose Escalation										Dose Expansion	Total (n = 38)
Category	5 mg capsule (*n* = 2)	10 mg capsule (*n* = 2)	20 mg capsule (*n* = 10)	30 mg capsule (*n* = 2)	45 mg capsule (*n* = 2)	45 mg tablet (*n* = 2)	80 mg tablet (*n* = 2)	100 mg tablet (*n* = 6)	120 mg tablet (*n* = 4)	Total (*n* = 32)	120 mg Tablet (*n* = 6)	
**Best overall response,*n* (%)**												
Complete response	0 (0.0)	0 (0.0)	0 (0.0)	0 (0.0)	0 (0.0)	0 (0.0)	0 (0.0)	0 (0.0)	0 (0.0)	**0 (0.0)**	0 (0.0)	0 (0.0)
Partial response	0 (0.0)	0 (0.0)	0 (0.0)	0 (0.0)	0 (0.0)	0 (0.0)	0 (0.0)	0 (0.0)	0 (0.0)	**0 (0.0)**	0 (0.0)	0 (0.0)
Stable disease	1 (50.0)	2 (100)	4 (40.0)	2 (100)	0 (0.0)	0 (0.0)	0 (0.0)	3 (50.0)	0 (0.0)	**12 (37.5)**	2 (33.3)	14 (36.8)
Durable stable disease^a^	0 (0.0)	1 (50.0)	3 (30.0)	1 (50.0)	0 (0.0)	0 (0.0)	0 (0.0)	1 (16.7)	0 (0.0)	**6 (18.8)**	0 (0.0)	6 (15.8)
Progressive disease	1 (50.0)	0 (0.0)	5 (50.0)	0 (0.0)	1 (50.0)	2 (100)	2 (100)	3 (50.0)	1 (25.0)	**15 (46.9)**	3 (50.0)	18 (47.4)
Unknown/not evaluable	0 (0.0)	0 (0.0)	1 (10.0)	0 (0.0)	1 (50.0)	0 (0.0)	0 (0.0)	0 (0.0)	3 (75.0)	**5 (15.6)**	1 (16.7)	6 (15.8)

E7386 was administered twice daily in all dose levels/formulations. Percentages are based on the total number of patients in the relevant column.

^a^Durable stable disease is a subset of stable disease with a duration of stable disease of ≥23 weeks from the first dose.

BOR of progressive disease was observed in 18 (47.4%) patients, 15 (46.9%) in the Escalation Part, and 3 (50%) in the Expansion Part.

One patient with a desmoid tumour (who received E7386 30 mg BID, was previously treated with vinorelbine, and had a BOR of progressive disease before enrolment in the study) is currently the only patient receiving study treatment; this patient has had SD for more than 6 years (over 5 years as of the data cutoff date), and their tumour harbours a *CTNNB1* mutation. To date, tumour shrinkage of up to 12–13% has been observed (Supplementary Fig. 4), in addition to changes in cellularity.

Median PFS was 1.9 months (95% CI: 1.8–3.5) in the overall population, 1.9 months (95% CI: 1.8–3.7) in the Escalation Part, and 1.8 months (95% CI: 1.4–not evaluable) in the Expansion Part. The overall PFS rate was 10.2% (95% CI: 2.6–23.7), 6.8% (95% CI: 1.2–19.3), 3.4% (95% CI: 0.3–14.7), and 3.4% (95% CI: 0.3–14.7) at 6, 12, 18, and 24 months, respectively.

## Discussion and conclusions

The aberrant activation of the Wnt/β-catenin signalling pathway is frequently observed in various advanced neoplasms, including HCC, and is strongly implicated in tumorigenesis, progression, and therapeutic resistance [5]. Despite various treatments showing efficacy in preclinical models, challenges exist regarding pathway complexity, interconnection with other signalling networks, and clinical translation; ongoing research and clinical trials aim to bring Wnt-targeted therapies to the forefront of HCC treatment [5].

In this first-in-human Phase 1 Study 101, we investigated the safety and tolerability profile of E7386 as a single agent administered orally in patients with selected advanced or recurrent neoplasms. E7386 was evaluated as monotherapy at doses ranging from 5 to 120 mg BID in 38 heavily pretreated patients with advanced or recurrent solid tumours (Escalation Part) or with *CTNNB1*-mutated HCC (Expansion Part).

The primary objectives of the study, to assess the safety/tolerability profile of E7386 and to determine the RP2D of E7386, were met. The RP2D of E7386, predominantly based on safety and tolerability results, was determined as 120 mg BID. One patient in the 20 mg BID capsule group (grade 2 lethargy and grade 2 decreased appetite) met the criteria of DLTs in the Escalation Part. Subsequently, dose escalation up to 120 mg BID resulted in no additional DLTs. Per protocol, the maximum dose to be tested was up to 140 mg BID; however, this was not evaluated due to the observed variability in PK exposure, which could potentially result in overlap between the 140 and 160 mg BID dose levels. Thus, the MTD of E7386 monotherapy was not reached. The RP2D of 120 mg BID of Study 101 is aligned with the RP2D determined in Study 103 (clinicaltrials.gov identifier: NCT03833700) [11], where E7386 monotherapy was evaluated in patients with advanced solid tumours in Japan, and 2 DLTs occurred in 2 patients tested at E7386 160 mg BID.

Overall, E7386 monotherapy had a manageable safety profile. The most common TRAEs were nausea (65.8%), vomiting (60.5%), fatigue (28.9%), decreased appetite, and diarrhoea (15.8% each). Patients were not recommended prophylactic treatment for nausea or vomiting during the DLT assessment period (which may explain their frequent incidence); however, nausea and vomiting were well controlled once 5-HT_3_ receptor antagonists were allowed. No TRAE of nausea or vomiting led to drug discontinuation. Overall, 7 (18.4%) patients experienced grade ≥3 TRAEs. Most TRAEs were gastrointestinal events of grades 1 and 2. Notably, gastrointestinal disorders and fatigue are commonly reported TRAEs with other agents acting on the Wnt pathway, including DKN-01 (fatigue: 35%; nausea: 35%) [16] and tegavivint (fatigue: 71%; nausea: 33%) [17].

Increasing E7386 plasma concentrations were observed after the administration of increasing doses of E7386. Overall, PK exposure, as measured by AUC and C_max_, appeared to increase with increasing dose, although large intersubject variability was observed. Excretion via urine was a minor pathway of E7386 elimination. These results were consistent with those reported in Study 103 after E7386 monotherapy [11].

Although no patients had an objective response in our study, a BOR of SD was observed in 14 (36.8%) patients, including SD lasting more than 23 weeks in 6 (18.8%) patients during the Escalation Part and in no patients in the Expansion Part. One patient with desmoid tumour and *CTNNB1* mutations treated with E7386 30 mg BID has had SD for more than 6 years now (over 5 years as of data cutoff date) and has shown maximum tumour shrinkage at just over 6 years. Correspondingly, in Study 103, 1 patient with a desmoid tumour and mutations in *APC* with familial adenomatous polyposis experienced SD lasting >23 weeks, followed by a partial response after 18 months of treatment; this patient was still receiving treatment as of the data cutoff date [11].

Thus, data suggest evidence of disease stabilisation with E7386 monotherapy, which is in line with findings from Study 103 [11] and studies of other monotherapies targeting the Wnt pathway in cancer, for which observed antitumour activity as monotherapy has also been limited [18–23]. In the Expansion Part, patients with HCC whose tumours harboured *CTNNB1* gain-of-function mutations were selected; however, defining the right population who may respond to Wnt modulators remains challenging, especially for those patients with HCC and treatment-refractory disease.

*CTNNB1* mutations define a distinctive subgroup of HCCs characterised by a well-differentiated phenotype, an immune-excluded microenvironment, and a low proliferative capacity (even if pathway modulation occurs), which together might explain the lack of radiologically quantifiable responses observed in this study [24]. Alterations of the Wnt/CTNNB1 pathway are associated with lower disease control rates to immunotherapy, highlighting how this population may be prognostically disadvantaged, irrespective of treatment [25].

Future studies focusing on molecular selection beyond the presence of *CTNNB1*-activating mutations may further clarify the appropriate HCC patient population for treatment with Wnt modulators.

Limitations of our study include a small cohort size (between 2 and 10) for different dose levels of E7386 across various tumour types, which limits the interpretation and generalisability of the data. The small sample size may have also limited the detection of less frequent adverse toxicities and the variability of the PK data.

Taken together, this first-in-human Phase 1 study indicates that the orally administered E7386 has a manageable safety profile with some evidence of disease stabilisation in this heavily pretreated heterogeneous population. The limited antitumor activity observed may reflect the biological complexity of Wnt-signalling. Early clinical data suggest a more promising role for E7386 in combination with other anticancer agents: a Phase 1b/2 study (NCT04008797) of E7386 in combination with the multikinase inhibitor, lenvatinib, has demonstrated manageable safety and promising antitumour activity in advanced endometrial cancer [26]; the dose-optimisation part in patients with endometrial cancer is currently in progress [27]. These findings underscore the need for refined biomarker-targeting strategies and a combinational approach to improve clinical benefit.

## Supplementary information

Supplement

## Data Availability

The data will not be available for sharing at this time as the data are commercially confidential. However, Eisai will consider written requests to share the data on a case-by-case basis.
