# Supplementary material for: A first-in-human, open-label multicentre Phase 1 study of the orally administered E7386 in patients with selected advanced neoplasms
Source: Br J Cancer. 2026 Jun 4;135(5):736–46. doi: 10.1038/s41416-026-03488-w (PMC13478614; doi:10.1038/s41416-026-03488-w)

Supplementary Information

Determination of Dose-Limiting Toxicity Analyses

Dose-limiting toxicities (DLTs) are any of the following drug-related toxicities (any toxicities considered related to E7386) occurring during cycle 1 as assessed by the investigator. DLT determination and dose escalation were agreed upon between investigators and the study sponsor; additional medical advice was sought if necessary. Grading of toxicities was based on the National Cancer Institute Common Terminology Criteria for Adverse Events (CTCAE) version 4.03.

- Nonhematologic toxicity grade ≥3, except diarrhea, nausea and vomiting unless lasting >3 days despite best/optimal supportive treatment.
- Nonhematologic laboratory findings of grade ≥3 that were grade ≤1 at baseline, with the exception of abnormal grade 3 laboratory values with no clinical significance that resolve within 7 days (this includes electrolyte abnormalities that respond to medical intervention).
- Hematologic toxicity:
    - Grade 4 neutropenia not resolving to grade 2 or less within 7 days, or grade 3 or 4 febrile neutropenia.
    - Grade 4 thrombocytopenia, or grade 3 thrombocytopenia with bleeding or lasting >7 days.
- Grade 2 nonhematologic toxicities related to E7386 treatment, which, in the opinion of the treating investigator, required a dose reduction or discontinuation of study drug, or led to the patient's failure to complete at least 21 days of study drug administration in cycle 1, were deemed to be dose-limiting if agreed upon by participating investigators and the Sponsor.
- Bone mineral density (BMD) change >5% (worsening) from baseline (non-CTCAE), despite at least 2 weeks of bisphosphonate or denosumab treatment (dose escalation).
- β-C-terminal telopeptide (β-CTX) increase >2-fold from baseline AND >1000 pg/mL (non-CTCAE), despite at least 2 weeks of bisphosphonate or denosumab treatment, or β-C-terminal telopeptide increase >60% from cycle 1 day 1.
- Grade 2 osteoporosis (BMD T-score < −2.5), despite 2 or more weeks of bisphosphonate or denosumab treatment.
- Grade 3 osteoporosis.

- Bone fragility fracture of any severity, defined as any fracture without a history of trauma or because of a fall from standing height or less (non-CTCAE).
- Any other toxicity assessed as related to E7386 treatment and which in the opinion of the study investigator(s) and the Sponsor medical monitor constitutes a DLT.
- Patients who had missed ≥7 days of dosing in cycle 1 due to drug-related toxicity (but not qualifying for a DLT) were assessed as experiencing a DLT.

In addition, all safety data were monitored on an ongoing basis including late-term toxicities such as bone toxicity, and were considered when evaluating DLTs for the purposes of making dose escalation decisions and before recommending a phase 2 dose for further evaluation. Patients who prematurely withdrew for reasons other than DLT, or failed to complete 75% of the planned total dose without a DLT in cycle 1, were replaced.

Supplementary Table 1. Treatment-emergent adverse events occurring in > 15% of patients overall in the Dose Escalation and Dose Expansion parts.

Category	Dose Escalation[a]										Dose Expansion[a]	Total (n = 38)
	5 mg Capsule (n = 2)	10 mg Capsule (n = 2)	20 mg Cap-sule (n = 10)	30 mg Capsule (n = 2)	45 mg Capsule (n = 2)	45 mg Tablet (n = 2)	80 mg Tablet (n = 2)	100 mg Tablet (n = 6)	120 mg Tablet (n = 4)	Total (n = 32)	120 mg Tablet (n = 6)	
**TEAEs, n (%)**	2 (100)	2 (100)	10 (100)	2 (100)	2 (100)	2 (100)	2 (100)	6 (100)	4 (100)	32 (100)	6 (100)	38 (100)
Nausea	1 (50.0)	2 (100)	8 (80.0)	2 (100)	2 (100)	1 (50.0)	2 (100)	5 (83.3)	3 (75.0)	26 (81.3)	4 (66.7)	30 (78.9)
Vomiting	0 (0.0)	2 (100)	6 (60.0)	2 (100)	1 (50.0)	1 (50.0)	1 (50.0)	3 (50.0)	3 (75.0)	19 (59.4)	5 (83.3)	24 (63.2)
Fatigue	0 (0.0)	1 (50.0)	3 (30.0)	2 (100)	0 (0.0)	1 (50.0)	0 (0.0)	3 (50.0)	1 (25.0)	11 (34.4)	4 (66.7)	15 (39.5)
Diarrhoea	0 (0.0)	0 (0.0)	0 (0.0)	2 (100)	0 (0.0)	0 (0.0)	1 (50.0)	2 (33.3)	3 (75.0)	8 (25.0)	3 (50.0)	11 (28.9)
Constipation	1 (50.0)	0 (0.0)	3 (30.0)	1 (50.0)	1 (50.0)	0 (0.0)	1 (50.0)	1 (16.7)	1 (25.0)	9 (28.1)	2 (33.3)	11 (28.9)
Decreased appetite	0 (0.0)	1 (50.0)	4 (40.0)	0 (0.0)	0 (0.0)	0 (0.0)	0 (0.0)	0 (0.0)	1 (25.0)	6 (18.8)	4 (66.7)	10 (26.3)
Anaemia	0 (0.0)	2 (100)	2 (20.0)	1 (50.0)	0 (0.0)	0 (0.0)	0 (0.0)	1 (16.7)	1 (25.0)	7 (21.9)	2 (33.3)	9 (23.7)
Abdominal pain	0 (0.0)	0 (0.0)	3 (30.0)	1 (50.0)	1 (50.0)	0 (0.0)	1 (50.0)	0 (0.0)	1 (25.0)	7 (21.9)	1 (16.7)	8 (21.1)
Increased aspartate aminotransferase	0 (0.0)	1 (50.0)	0 (0.0)	1 (50.0)	0 (0.0)	0 (0.0)	1 (50.0)	0 (0.0)	2 (50.0)	5 (15.6)	3 (50.0)	8 (21.1)
Decreased weight	0 (0.0)	1 (50.0)	2 (20.0)	0 (0.0)	0 (0.0)	0 (0.0)	0 (0.0)	0 (0.0)	1 (25.0)	4 (12.5)	3 (50.0)	7 (18.4)
**TEAE: worst CTCAE grade of ≥ 3, n (%)**	1 (50.0)	1 (50.0)	7 (70.0)	1 (50.0)	0 (0.0)	0 (0.0)	1 (50.0)	1 (16.7)	4 (100.0)	16 (50.0)	3 (50.0)	19 (50.0)
**Serious TEAEs,[a] n (%)**	0 (0.0)	2 (100.0)	6 (60.0)	0 (0.0)	0 (0.0)	0 (0.0)	1 (50.0)	0 (0.0)	4 (100.0)	13 (40.6)	1 (16.7)	14 (36.8)
**TEAEs leading to study drug discontinuation, n (%)**	0 (0.0)	0 (0.0)	2 (20.0)	0 (0.0)	1 (50.0)	0 (0.0)	0 (0.0)	0 (0.0)	3 (75.0)	6 (18.8)	1 (16.7)	7 (18.4)
**TEAEs leading to study drug dose reduction, n (%)**	0 (0.0)	0 (0.0)	1 (10.0)	0 (0.0)	0 (0.0)	0 (0.0)	0 (0.0)	0 (0.0)	0 (0.0)	1 (3.1)	2 (33.3)	3 (7.9)
**TEAEs leading to study drug interruption, n (%)**	0 (0.0)	1 (50.0)	5 (50.0)	1 (50.0)	0 (0.0)	0 (0.0)	1 (50.0)	1 (16.7)	3 (75.0)	12 (37.5)	3 (50.0)	15 (39.5)

CTCAE, Common Terminology Criteria for Adverse Events; TEAE, treatment-emergent adverse event; TRAE, treatment-related adverse event.
[a]Patients may be counted in multiple categories.

Supplementary Fig. 1. Design of phase 1 Study 101. [a]The initial dose was 5 mg twice daily for capsules up to 45 mg twice daily (5 mg, 10 mg, 20 mg, 30 mg, and 45 mg twice daily) then the formulation was switched from capsule to tablet (immediate release) starting from 45 mg twice daily (45 mg, 80 mg, 100 mg, and 120 mg twice daily), continuously in 28-day cycles, as the capsule formulation showed inconsistent absorption and very high pharmacokinetic variability across all tested dose levels. BCLC, Barcelona Clinic Liver Cancer; BID, twice daily; ctDNA, circulating tumour DNA; DLT, dose-limiting toxicity; ECOG, Eastern Cooperative Oncology Group; HCC, hepatocellular carcinoma; PS, performance status; RP2D, recommended phase 2 dose.

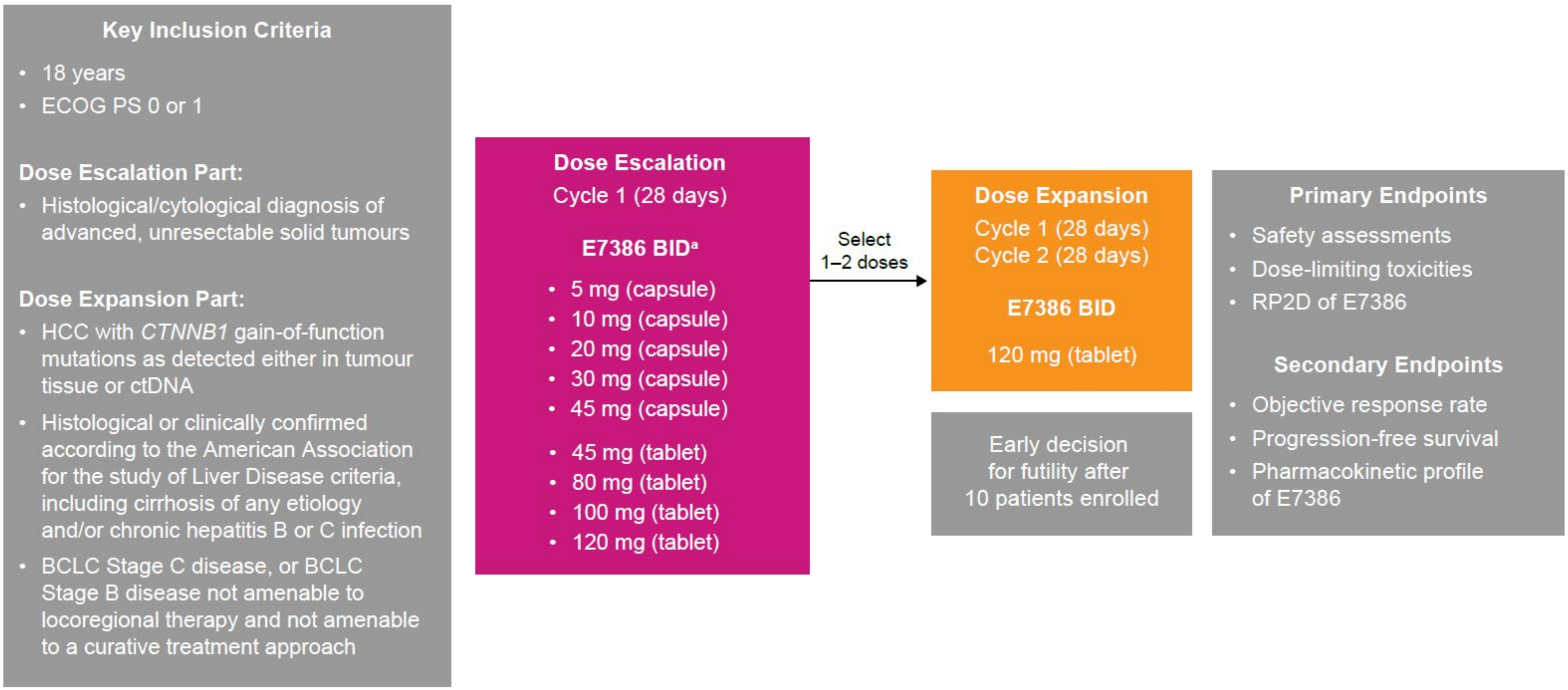

Supplementary Fig. 2. Patient enrolment and disposition. [a]Based on the primary reason reported on the screening disposition case report form.

[b]Percentages are based on the total number of patients in the Full Analysis set within the relevant study part. DCO, data cutoff.

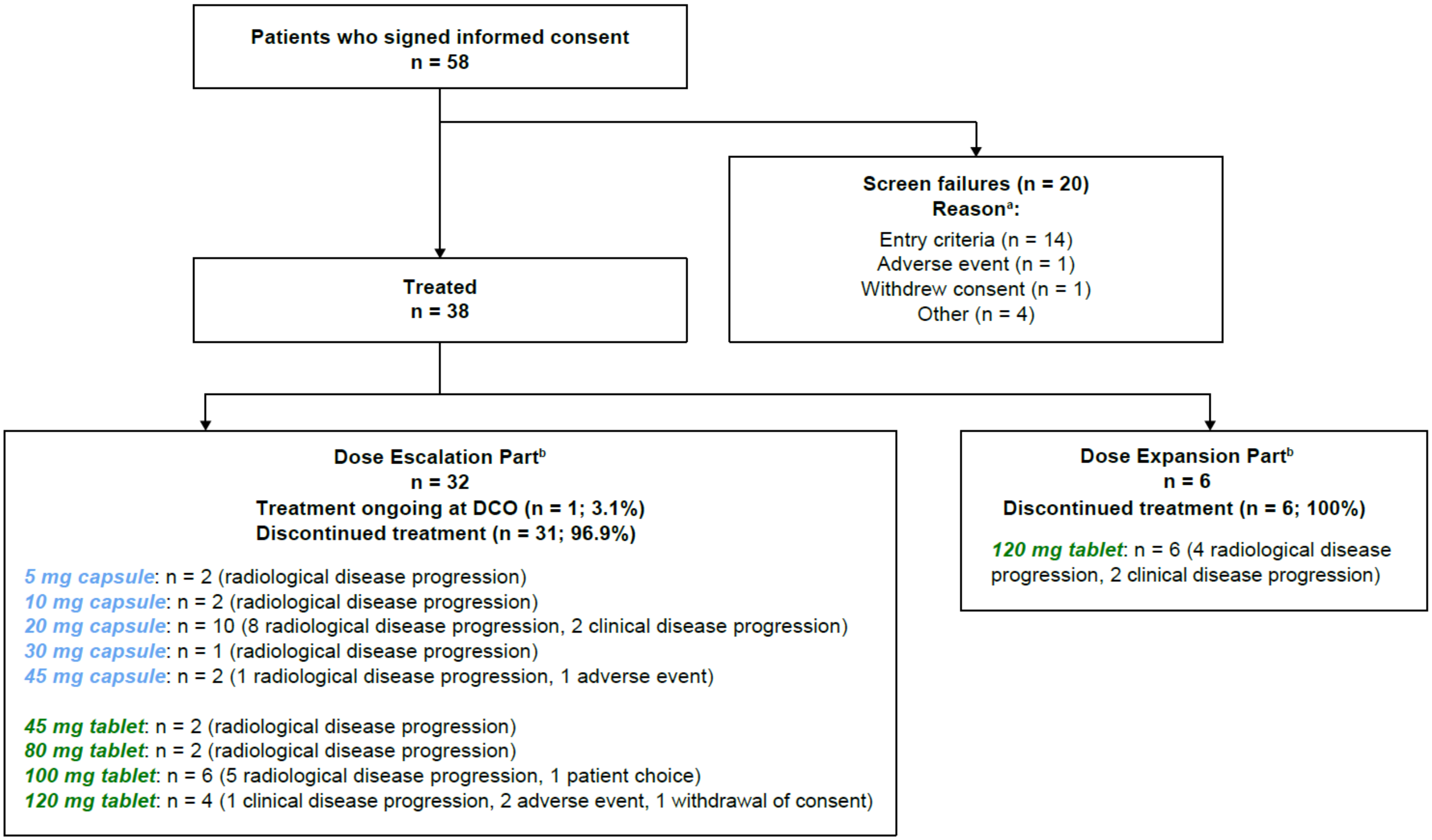

Supplementary Fig. 3. Linear plot of mean (+SD) E7386 plasma concentration. BID, twice daily; SD, standard deviation.

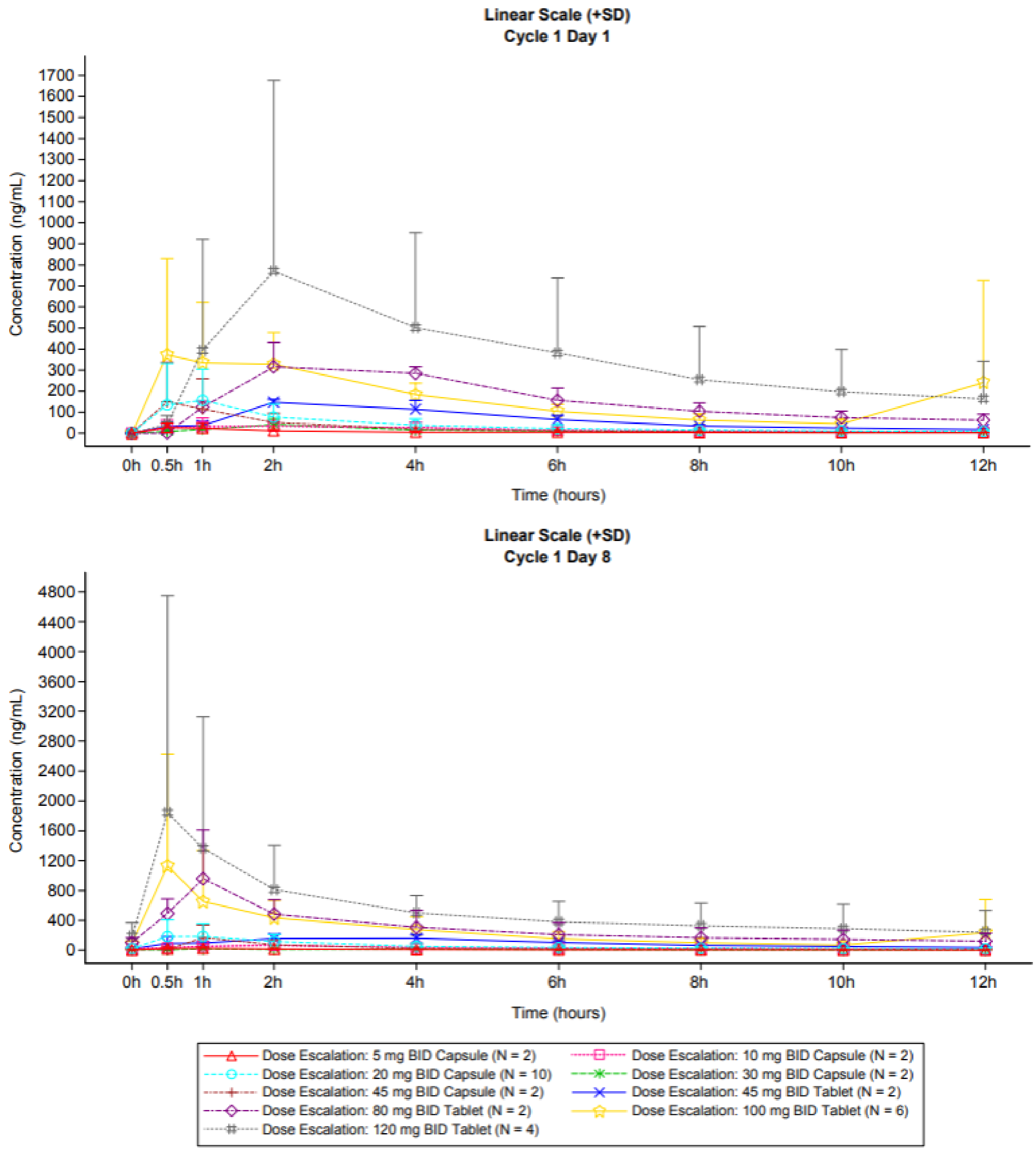

Supplementary Fig. 4. Magnetic resonance imaging of the patient with desmoid tumour who has had stable disease on study treatment for more than 6 years.

Coronal T2 MRI (top row) images at baseline (January 2019), May 2024 and most recent (October 2025) in a 65 year old patient with right shoulder desmoid tumour supplemented by axial DWI sequences showing a reduction in size (12%) and increase (26%) in ADC values of the lesion in keeping with reduction in tumour cellularity (bottom row). The lesion is causing less distortion of the overlying subcutaneous tissues (white arrow) and has become rounder in morphology suggestive of a reduction in desmoid reaction.

ADC, apparent diffusion coefficient; DWI, diffusion weighted imaging; MRI, magnetic resonance imaging; T2-W, T2-weighted.

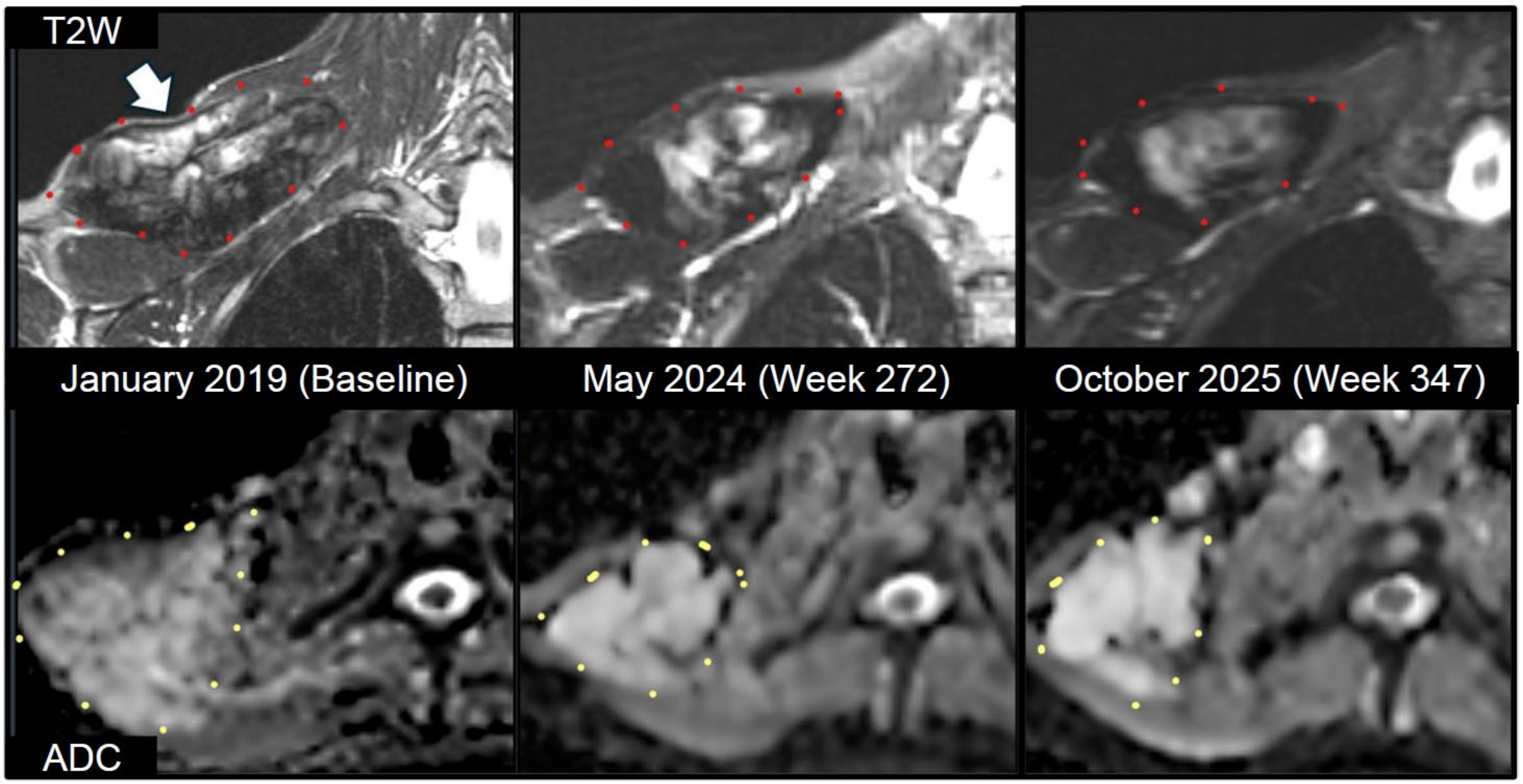

Supplement: Supplementary file 1 [file 41416_2026_3488_MOESM1_ESM.pdf]
